# An interpretable machine learning model for predicting symptomatic pelvic lymphocele after pelvic lymphadenectomy in cervical cancer

**DOI:** 10.3389/fonc.2026.1754363

**Published:** 2026-04-15

**Authors:** Yiyue Wang, Nenghuan Tang, Weimin Zhang, Yuhua Zeng

**Affiliations:** Department of Obstetrics and Gynecology, Affiliated Hospital of North Sichuan Medical College, Nanchong, Sichuan, China

**Keywords:** cervical cancer, machine learning model, pelvic lymphadenectomy, SHapley additive exPlanations method, symptomatic pelvic lymphocele

## Abstract

**Objective:**

To develop and validate an interpretable machine learning model using retrospective analysis of clinical characteristics and laboratory data from patients with cervical cancer to predict symptomatic pelvic lymphocele (SPL) following pelvic lymphadenectomy.

**Methods:**

Clinical data were collected from 221 patients with cervical cancer who underwent pelvic lymphadenectomy at the Affiliated Hospital of North Sichuan Medical College between January 2023 and September 2024. SPL occurred in 44 patients, whereas 177 had asymptomatic lymphoceles or no lymphocele. Univariate analysis identified risk factors for SPL. Four predictive models including Logistic Regression (LR), K–Nearest Neighbors (KNN), Gradient Boosting Machine (GBM), and Extreme Gradient Boosting (XGBoost) were developed. Model performance was assessed using the Area Under the Receiver Operating Characteristic Curve (AUC), Decision Curve Analysis (DCA), and Brier Score. The SHapley Additive exPlanations (SHAP) method was applied to determine feature importance.

**Results:**

The incidence of SPL among patients with cervical cancer was 19.9%. Among the four models, the KNN model demonstrated relatively better predictive performance in both the training and test sets (training set AUC = 0.952, 95% confidence interval (CI) 0.924–0.980; test set AUC = 0.832, 95% CI 0.692–0.972; Brier Score = 0.118). DCA indicated favorable clinical utility for the KNN model. SHAP analysis identified the most predictive features for SPL as diabetes, surgical approach, preoperative monocyte-to-lymphocyte ratio, preoperative fibrinogen level, and tumor size.

**Conclusion:**

This interpretable model identified key features associated with SPL following pelvic lymphadenectomy in patients with cervical cancer, yielding preliminary insights that support hypothesis generation for future research.

## Introduction

Radical hysterectomy with pelvic lymphadenectomy is a commonly used surgical treatment for cervical cancer (1–3). Common postoperative complications include lymphedema and pelvic lymphocele (4, 5). Lymphoceles usually appear within 3–8 weeks after surgery (6), however they can occasionally develop more than 1 year postoperatively (4). The reported incidence of lymphocele after pelvic lymphadenectomy for gynecologic malignancies ranges from 8% to 63% (7). Most lymphoceles are asymptomatic and resolve spontaneously without intervention (8). However, 3–34.5% develop into symptomatic pelvic lymphocele (SPL) (4, 7, 9–12), which can cause pelvic pain, infection, lower urinary tract symptoms, leg edema, hydronephrosis with renal impairment, deep vein thrombosis, and other complications related to compression of nearby structures (5, 6, 8). SPL often requires intervention, is challenging to manage, prolongs hospitalization, delays adjuvant therapy (13), and significantly affects long-term quality of life. Therefore, identifying high-risk factors for SPL and optimizing preoperative and intraoperative decision-making are essential.

Previous studies have suggested associations between SPL after gynecologic oncology surgery and variables such as smoking, body mass index (BMI), hypertension, prophylactic subcutaneous heparin use, surgical technique, number of lymph nodes removed, lymph node metastasis, and adjuvant radiotherapy (8, 14, 15), though findings have been inconsistent. Research focusing on cervical cancer populations has identified associations only with smoking, BMI, and prior abdominal surgery (14). Current studies on SPL among patients with cervical cancer remain limited and inconclusive. Recently, machine learning models have shown notable advantages and have been increasingly applied in disease prediction (16, 17). Therefore, we retrospectively collected clinical data from patients with cervical cancer at our institution to develop and validate multiple machine learning models aimed at predicting SPL occurrence.

## Materials and methods

### Study population

Clinical data were retrospectively collected from 224 patients with cervical cancer who underwent radical hysterectomy with pelvic lymphadenectomy at the Affiliated Hospital of North Sichuan Medical College between January 2023 and September 2024. The study was approved by the hospital’s Ethics Committee (2025ER377-1).

The inclusion criteria for this study were as follows: 1) Patients with cervical cancer treated at our hospital with radical hysterectomy and pelvic lymphadenectomy, with or without para-aortic lymphadenectomy. 2) Histopathologically confirmed cervical cancer (according to the 2018 International Federation of Gynecology and Obstetrics (FIGO) staging system). 3) Complete medical records. The exclusion criteria were as follows: 1) History of previous pelvic/abdominal lymph node resection or infection. 2) Concurrent other malignant tumors. 3) Fewer than one follow-up visit at our hospital within 1 year postoperatively.

### Definitions

*Pelvic lymphocele* was defined as a newly developed unilocular or multilocular cystic structure at the lymphadenectomy site containing fluid visible on follow-up imaging such as computed tomography (CT), magnetic resonance imaging (MRI), or ultrasound.

*SPL* was defined as radiologically confirmed lymphocele associated with symptoms attributable to the lesion, such as lower abdominal pain or discomfort, lower limb edema, secondary infection (fever, worsened abdominal pain), hydronephrosis, or deep vein thrombosis.

### Surgery and follow-up

All surgeries were performed by our hospital’s surgical team. All patients received prophylactic antibiotics. Postoperative follow-up, including gynecologic examination and ultrasound, was conducted according to National Comprehensive Cancer Network (NCCN) guidelines and patient needs, typically at 1, 3, 6, and 12 months postoperatively.

### Study variables and data extraction

After screening 224 patients, 3 were excluded due to insufficient follow-up. Data from 221 patients were analyzed, including:

*Demographics:* Age, BMI.

*Medical History and Comorbidities:* Previous abdominal surgery, diabetes, hypertension.

*Tumor Characteristics:* Pathological type (squamous vs. non-squamous), FIGO stage, tumor size, depth of invasion (> 1/2 myometrium), preoperative lymph node enlargement, and lymph node metastasis.

*Treatment Details:* Surgical approach (open vs. Laparoscopic), oophorectomy, para-aortic lymphadenectomy, number of lymph nodes removed, operative time, intraoperative blood loss, neoadjuvant chemotherapy, neoadjuvant radiotherapy, neoadjuvant chemoradiotherapy, adjuvant chemotherapy, adjuvant radiotherapy, adjuvant chemoradiotherapy, pelvic drainage method (transabdominal pelvic drainage vs. transvaginal T-tube drainage), and drainage duration (removal criterion: 24-hour output< 50 mL) (18, 19).

*Laboratory Results:* Preoperative fibrinogen (FIB), hemoglobin, platelets, monocytes, neutrophils, and lymphocytes count, neutrophil-to-lymphocyte ratio (NLR), monocyte-to-lymphocyte ratio (MLR), and postoperative albumin.

**Figure 1:**
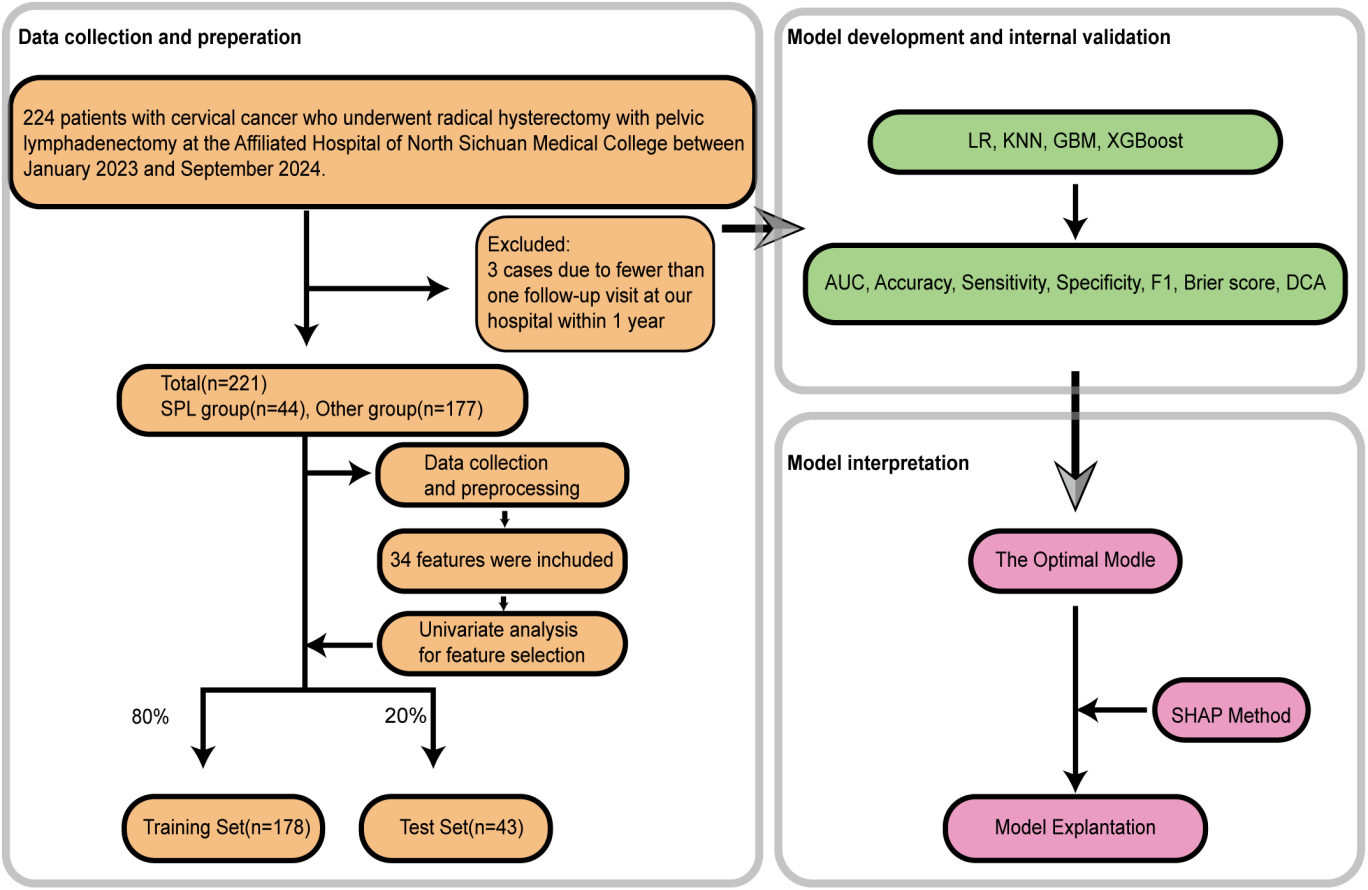
The flowchart of the study.

BMI = weight (kg)/height (m²). NLR = Neutrophil count (10^9^/L)/Lymphocyte count (10^9^/L). MLR = Monocyte count (10^9^/L)/Lymphocyte count (10^9^/L). Tumor type, stage, and lymph node status were determined from final postoperative histopathology. The study flowchart is shown in **Figure 1**.

### Statistical analysis

Analyses were conducted using R software. Variables with extreme class imbalance or substantial missing values were excluded from further analysis. Specifically, smoking status was excluded due to very low prevalence, and preoperative albumin was excluded due to a high missing rate. Missing data were handled with mean imputation (**Supplementary Tables 1**-**3**; **Supplementary Figure 1**). Normally distributed continuous variables are expressed as mean ± standard deviation and were compared using the t-test. Non-normally distributed data are presented as median and interquartile range and were compared using the Mann–Whitney U test. Categorical variables are presented as frequencies (n [%]) and compared using the chi-square or Fisher’s exact test, as appropriate. A P-value<0.05 was considered statistically significant.

### Predictive model construction and evaluation

Variables with P<0.05 in univariate analysis were used to develop predictive models in R software (version 4.4.2). Four models were constructed: Logistic Regression (LR) (20), K-Nearest Neighbors (KNN) (21), Gradient Boosting Machine (GBM) (22), and Extreme Gradient Boosting (XGBoost) (23). LR, an established and interpretable binary classification algorithm, served as the baseline. KNN, a non-parametric method, performs well in small datasets and captures complex nonlinear relationships. GBM and XGBoost, ensemble decision tree algorithms, offer high predictive accuracy and effectively address data imbalance. Model discrimination was assessed using the Area Under the ROC Curve (AUC). Decision Curve Analysis (DCA) (24) evaluated clinical utility, and the Brier Score (25) measured the accuracy of probability predictions. To address the “black box” nature of machine learning models, SHapley Additive exPlanations (SHAP) analysis (26) was employed to interpret model results and assess feature importance by quantifying each feature’s contribution to the prediction.

## Results

Among 224 patients with cervical cancer who underwent pelvic lymphadenectomy, 3 were lost to follow-up. Symptomatic lymphocele occurred in 44 patients (SPL group), while 177 patients had asymptomatic lymphoceles or no lymphocele (Other group), yielding an SPL incidence of 19.9%. The flow diagram of patient selection and grouping is presented in **Figure 1**. In the SPL group, symptoms included lower abdominal pain (8 patients), inguinal pain (3), lower back pain (2), lymphocele infection (13), hydronephrosis (34), lower limb edema (8), and lower limb deep vein thrombosis (5) (data not shown).

### Univariate analysis results

Among 34 analyzed features, univariate analysis revealed that patients in the SPL group had a significantly higher prevalence of diabetes, larger tumor diameter (> 4 cm), higher FIB levels, higher MLR, and a greater proportion underwent open surgery (all P<0.05). No significant differences were observed for other variables (all P > 0.05) (**Table 1**). Baseline characteristics of the training set and test set are presented in **Supplementary Table 4**.

**Table 1:** Univariate analysis of symptomatic pelvic lymphocele following pelvic lymphadenectomy in patients with cervical cancer.

Variables	Total (n=221)	SPL (n=44)	Other (n=177)	P
Age (mean (SD))	53.82 (9.33)	55.57 (9.83)	53.39 (9.18)	0.166
BMI (mean (SD))	23.71 (3.14)	23.65 (2.63)	23.72 (3.26)	0.883
Previous abdominal surgery = Yes (%)	55 (24.9)	11 (25.0)	44 (24.9)	1
Diabetes = Yes (%)	18 (8.1)	8 (18.2)	10 (5.6)	**0.016**
Hypertension = Yes (%)	34 (15.4)	8 (18.2)	26 (14.7)	0.733
Pathological type = non-squamous (%)	36 (16.3)	3 (6.8)	33 (18.6)	0.094
FIGO stage > IIA2 (%)	46 (20.8)	9 (20.5)	37 (20.9)	1
Tumorsize > 4cm (%)	35 (15.8)	12 (27.3)	23 (13.0)	**0.037**
Depth of invasion = > 50%myometrium (%)	11 (5.0)	3 (6.8)	8 (4.5)	0.81
preoperative lymph node enlargement = Yes (%)	41 (18.6)	12 (27.3)	29 (16.4)	0.148
Lymph node metastasis = Yes (%)	44 (19.9)	10 (22.7)	34 (19.2)	0.755
Surgical approach = Laparoscopic (%)	82 (37.1)	9 (20.5)	73 (41.2)	**0.017**
Oophorectomy = Yes (%)	204 (92.3)	42 (95.5)	162 (91.5)	0.576
Para-aortic lymphadenectomy = Yes (%)	29 (13.1)	8 (18.2)	21 (11.9)	0.389
Number of lymph nodes removed (mean (SD))	19.29 (8.46)	19.64 (8.01)	19.20 (8.59)	0.762
Operative time (mean (SD))	4.34 (1.13)	4.46 (1.25)	4.32 (1.10)	0.459
Intraoperative blood loss (mean (SD))	360.45 (265.27)	425.91 (270.14)	344.18 (262.29)	0.067
Neoadjuvant chemotherapy = Yes (%)	34 (15.4)	8 (18.2)	26 (14.7)	0.733
Neoadjuvan tadiotherapy = Yes (%)	21 (9.5)	7 (15.9)	14 (7.9)	0.183
Neoadjuvant chemoradiotherapy = Yes (%)	14 (6.3)	6 (13.6)	8 (4.5)	0.061
Adjuvant chemotherapy = Yes (%)	87 (39.4)	21 (47.7)	66 (37.3)	0.273
Adjuvant radiotherapy = Yes (%)	46 (20.8)	8 (18.2)	38 (21.5)	0.785
Adjuvant chemoradiotherapy = Yes (%)	9 (4.1)	1 (2.3)	8 (4.5)	0.804
Pelvic drainage method = Transabdominal (%)	172 (77.8)	37 (84.1)	135 (76.3)	0.36
Drainage duration (mean (SD))	5.09 (3.55)	5.89 (5.10)	4.89 (3.04)	0.095
PreoperativeFib (mean (SD))	3.55 (0.80)	3.78 (0.85)	3.49 (0.78)	**0.029**
PreoperativeHb (mean (SD))	126.08 (14.69)	124.30 (17.24)	126.52 (14.01)	0.37
PreoperativePlt (mean (SD))	218.11 (66.84)	221.18 (48.19)	217.35 (70.82)	0.734
PreoperativeMo (mean (SD))	0.32 (0.12)	0.34 (0.10)	0.31 (0.13)	0.204
PreoperativeNeu (mean (SD))	3.87 (1.25)	3.78 (1.33)	3.90 (1.23)	0.569
PreoperativeLy (mean (SD))	1.61 (0.53)	1.50 (0.57)	1.63 (0.51)	0.141
PreoperativeNLR (mean (SD))	2.63 (1.15)	2.84 (1.42)	2.58 (1.07)	0.166
PreoperativeMLR (mean (SD))	0.22 (0.11)	0.27 (0.16)	0.20 (0.09)	**0.001**
PostoperativeAlb (mean (SD))	33.58 (3.52)	33.11 (3.22)	33.69 (3.59)	0.327

Fib (fibrinogen), Hb (hemoglobin), Plt (platelets), Mo (monocytes), Neu (neutrophils), Ly (lymphocytes), NLR (neutrophil-to-lymphocyte ratio), MLR (monocyte-to-lymphocyte ratio), Alb (albumin).

Bolded numbers highlight variables that reached statistical significance ( P<0.05) in the univariate analysis.

### Model development and validation

**Figure 2:**
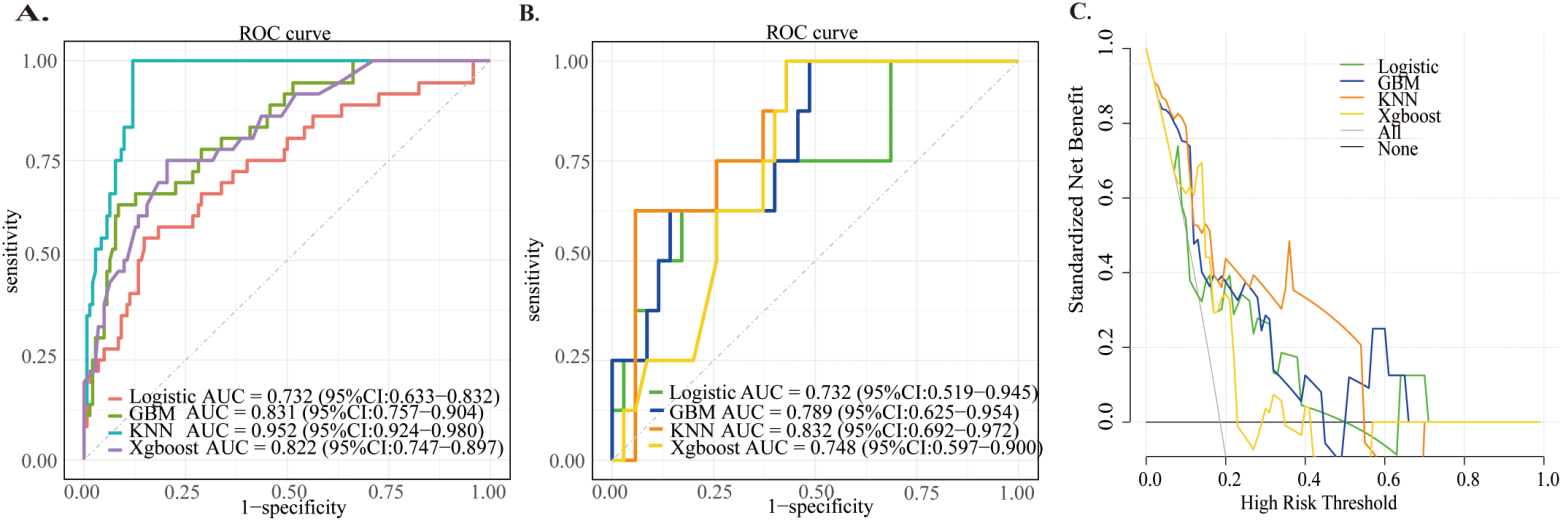
ROC curves and decision curves for four models: **(a)**. Training set ROC, **(b)**. Test set ROC, **(c)**. Test set DCA.

All participants were randomly divided into training and test sets in an 8:2 ratio. Using predictors identified in the univariate analysis, four machine learning models (LR, KNN, GBM, and XGBoost) were constructed with the training set and internally validated with the test set. The KNN model demonstrated relatively better predictive performance in both sets (**Figures 2a, b**; **Table 2**), with a training set AUC = 0.952 (95% CI: 0.924–0.980) and a test set AUC = 0.832 (95% CI: 0.692–0.972), and a Brier Score of 0.118. DCA results showed that the KNN model provided relatively higher net benefit across most threshold probabilities, indicating good clinical utility (**Figure 2c**). The KNN model exhibits a standout strength in sensitivity, which guarantees that no positive cases are overlooked. In tandem with this strong sensitivity, the model’s specificity is more modest (0.571)—but this performance balance aligns with the practical tradeoffs that are typically accepted for tools meant for initial risk assessment. It also matches our focus: framing the KNN model as a valuable initial tool to identify patients who might be at risk of symptomatic pelvic lymphocele. The overall performance of each model is summarized in **Table 2**.

**Table 2:** Performance of four models.

Model	Logistic	KNN	GBM	Xgboost
Accuracy	0.791	0.651	0.605	0.651
Sensitivity	0.625	1	1	1
Specificity	0.829	0.571	0.514	0.571
F1	0.526	0.516	0.485	0.516
BrierScore	0.136	0.118	0.133	0.147
Train_AUC(95%CI)	0.732(0.633-0.832)	0.952(0.924-0.980)	0.831(0.757-0.904)	0.822(0.747-0.897)
Test_AUC(95%CI)	0.732(0.519-0.945)	0.832(0.692-0.972)	0.789(0.625-0.954)	0.748(0.597-0.900)

AUC (the Area Under the ROC Curve), LR (Logistic Regression), KNN (K-Nearest Neighbors), GBM (Gradient Boosting Machine), XGBoost (Extreme Gradient Boosting).

### SHAP analysis and interpretation

**Figure 3:**
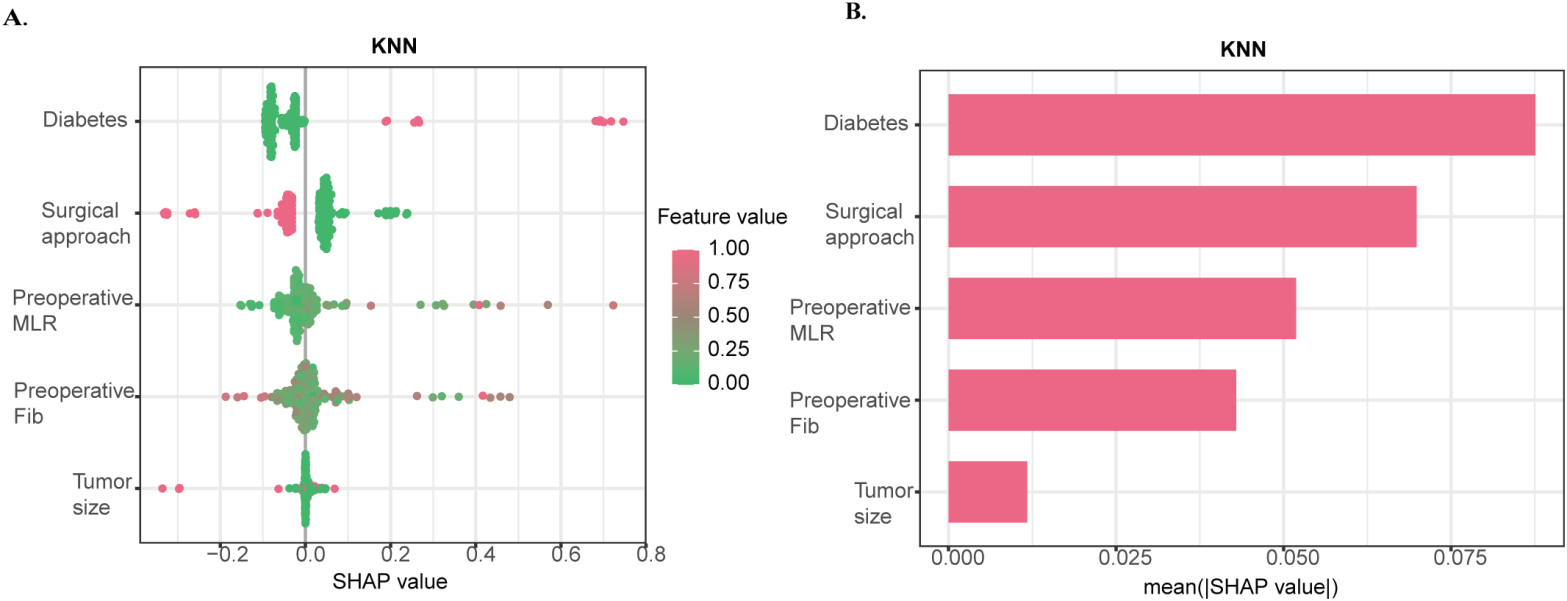
SHAP interpretation of the KNN Model. **(a)**. Each point in the beeswarm plot represents a patient; rows represent features; the x-axis shows SHAP values (positive values increase SPL risk, negative values decrease it); and color denotes the feature value (pink = high, green = low). **(b)**. Ranking of variable importance based on average SHAP values.

**Figure 4:**
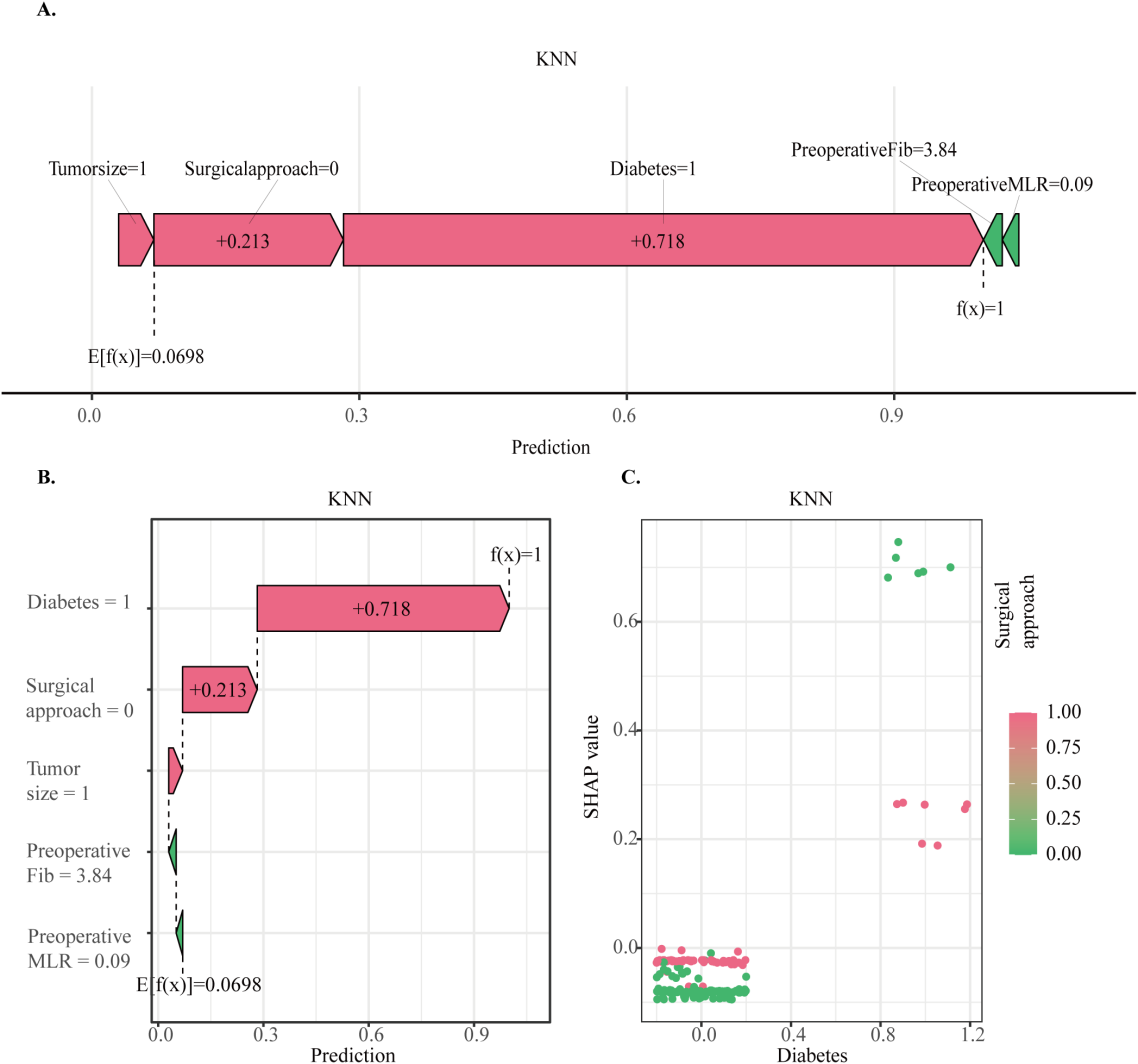
**(a, b)**. SHAP predictions for SPL. For categorical variables, Diabetes = 1 indicates diabetes; Surgical Approach = 0 indicates open surgery; Tumor Size = 1 indicates > 4 cm. **(c)**. Dependency plot illustrating relationships among the two variables with the highest SHAP values.

SHAP analysis was applied to the optimally performing KNN model to visualize features with predictive relevance to SPL (**Figure 3a**). Features were ranked by their relative predictive contribution to the model’s output for SPL as follows: presence of diabetes, surgical approach, MLR, FIB, and tumor size. The bar chart quantifies the mean absolute SHAP value for each feature (**Figure 3b**). A representative prediction case from the SPL group is shown in (**Figure 4a**), while (**Figure 4b**) illustrates each variable’s contribution to that patient’s SPL prediction. (**Figure 4c**) displays the dependency plot between the two most influential variables.

## Discussion

Reported incidence rates of SPL after gynecologic oncology surgery range from 3% to 34.5% (4, 7, 9–12). In this study, 44 of 221 patients with cervical cancer developed SPL, yielding an incidence of 19.9%, consistent with prior reports. Univariate analysis identified five risk factors: surgical approach, diabetes, tumor size, preoperative fibrinogen level, and preoperative MLR. The dataset was randomly divided 80:20 into training and test sets, and four machine learning models (LR, KNN, GBM, XGBoost) were constructed. The KNN model achieved relatively optimal predictive performance (training AUC 0.952, test AUC 0.832). SHAP analysis visualized the model’s predictive performance and ranked the features with the greatest predictive contribution to SPL as diabetes, surgical approach, MLR, FIB, and tumor size—identifying these as key model-associated features of SPL, with the analysis solely reflecting their relative contribution to the model’s predictive ability.

The exact mechanism of pelvic lymphocele formation post-lymphadenectomy remains incompletely understood. It is primarily attributed to disruption of lymphatic pathways and inadequate sealing of lymphatic channels (27). Significant lymphatic damage impairs normal drainage, leading to lymph accumulation within the pelvis or para-aortic region and lymphocele formation. Another hypothesis suggests that obstruction of the lymphatic system decreases reabsorption and increases intraluminal pressure, resulting in dilation and cyst formation (28). Some propose an imbalance between lower limb and pelvic lymph flow post-lymphadenectomy, promoting accumulation in the retroperitoneal space (29). Minor leakage typically causes small, asymptomatic cysts, whereas excessive leakage produces large or rapidly expanding lymphoceles that compress adjacent structures, causing pelvic pain, bloating, urinary frequency, constipation, edema, hydronephrosis, venous thrombosis, or infection. In patients with bone marrow suppression from adjuvant therapy, infected lymphoceles may progress to abscess, sepsis, or septic shock (5, 6, 8).

This study found that SPL occurred more frequently in patients with diabetes. Hyperglycemia induces vascular dysfunction preceding structural damage. Chronic plasma protein exudation and deposition on microvessel walls, coupled with impaired local microcirculation, metabolic accumulation, and inflammation, result in tissue hypoxia and malnutrition. These factors may increase lymphatic permeability and alter endothelial junctions, heightening the risk of postoperative lymph leakage and symptomatic compression (30). Hyperglycemia also suppresses immune function (e.g., impaired neutrophil migration, reduced phagocytosis, and bactericidal capacity) (31, 32), hindering wound healing and possibly explaining higher infection rates in diabetic patients with lymphoceles.

Open surgery was associated with a greater likelihood of SPL than laparoscopy, consistent with most studies (5, 33). The difference likely reflects the extent of lymphatic injury. Open procedures have a limited visual field, making precise identification and closure of lymphatics challenging. Frequent use of monopolar electrocautery or blunt dissection may fail to seal fine lymphatic vessels, leading to postoperative leakage. High thermal energy can also liquefy sealed lymphatics, increasing SPL risk. In contrast, laparoscopy offers a magnified view, and instruments like bipolar electrocautery or ultrasonic devices enable precise dissection and more effective lymphatic closure. Because lymphatic walls lack smooth muscle and lymph lacks platelets, these devices can seal connective tissue surrounding the lymphatics, enhancing closure and reducing postoperative lymph accumulation and SPL risk (34, 35).

Larger tumor diameter (> 4 cm) was also associated with a higher SPL rate, possibly due to increased surgical difficulty and incomplete lymphatic closure. B cells in draining lymph nodes secrete VEGF-A, promoting intranodal lymphangiogenesis (36), which may increase lymph flow and leakage when disrupted. Tumor-related changes in the microenvironment may further enhance lymphangiogenesis. Thus, removing these lymph nodes without adequate sealing of the rich lymphatic network can cause symptomatic cysts and related complications. Kelei et al. reported that larger cervical tumors correlate with higher fibrinogen levels (37), supporting our finding linking SPL to elevated fibrinogen. This may relate to coagulation and fibrinolysis abnormalities in patients with cervical cancer (38). Coagulation activation, fibrin deposition, and fibrinolysis in the tumor stroma can promote tumor growth and metastasis (39). A higher preoperative MLR was also linked to SPL, potentially reflecting cancer-associated inflammation. Patients with cancer often exhibit abnormal blood cell counts, including increased fibrinogen, neutrophils, platelets, and monocytes, along with reduced lymphocytes (40). A high MLR reflects relative lymphocytopenia or monocytosis. Lymphocytes are critical for antitumor defense, whereas monocytes are associated with poorer prognosis. Immunosuppression may increase infection risk within lymphoceles. Monocytes recruited into tumor tissue differentiate into tumor-associated macrophages (TAMs), promoting angiogenesis, matrix breakdown, and tumor motility, and producing compounds from mutagenic oxygen and nitrogen radicals to angiogenic factors, facilitating cancer progression (41, 42). We speculate that FIB and MLR promote tumor proliferation and lymphangiogenesis by modulating the tumor microenvironment. Enhanced lymphatic density around the tumor increases postoperative lymph leakage, raising SPL risk in patients with cervical cancer. Concurrent lymphocytopenia and immunosuppression may further predispose to infection.

Therefore, to reduce SPL incidence, strict preoperative blood glucose control is essential. Patients with large tumors, elevated fibrinogen, or high MLR require particular attention. Individualized surgical strategies should aim for optimal lymphatic closure to minimize postoperative complications.

Previous studies have associated SPL with smoking, prior abdominal surgery, BMI, hypertension, prophylactic heparin use, number of lymph nodes removed, lymph node metastasis, and adjuvant radiotherapy (12, 14, 15). Smoking was not analyzed due to low prevalence in this cohort, and postoperative heparin administration was routine, potentially introducing bias. Other variables showed no significant differences in univariate analysis, possibly due to the limited sample size or unequal group distribution. A primary limitation of the present study is the absence of external validation: all model performance assessments were based solely on internal validation using a random split of single-center study data, and the results are only exploratory findings from a small sample. This approach is unable to fully confirm the generalizability of the model, meaning its reliability thus requires further validation in large-sample, multi-center external cohorts.

This study preliminarily explores the association between inflammatory markers and SPL, contributing to understanding of lymphatic microcirculation in malignancy. The analyzed variables are readily obtainable from standard preoperative or postoperative assessments, imposing no additional patient burden and enhancing clinical applicability. Using SHAP for model visualization and feature ranking helps mitigate the “black box” issue of machine learning models.

## Conclusion

The incidence of symptomatic lymphocele after pelvic lymphadenectomy in patients with cervical cancer was 19.9%. A feature-selected KNN model demonstrated favorable predictive performance for SPL in internal validation analyses, and SHAP analysis quantified feature contributions, ranking importance as diabetes, open surgical approach, higher preoperative MLR, higher preoperative fibrinogen level, and tumor diameter > 4 cm. These findings identify potential key predictors of SPL and provide a foundation for future investigative work.

## Data Availability

The original contributions presented in the study are included in the article/**Supplementary Material**. Further inquiries can be directed to the corresponding author.
