# Supplementary figures and images for: An interpretable machine learning model for predicting symptomatic pelvic lymphocele after pelvic lymphadenectomy in cervical cancer
Source: Front Oncol. 2026 Apr 15;16:1754363. doi: 10.3389/fonc.2026.1754363 (PMC13125007; doi:10.3389/fonc.2026.1754363)

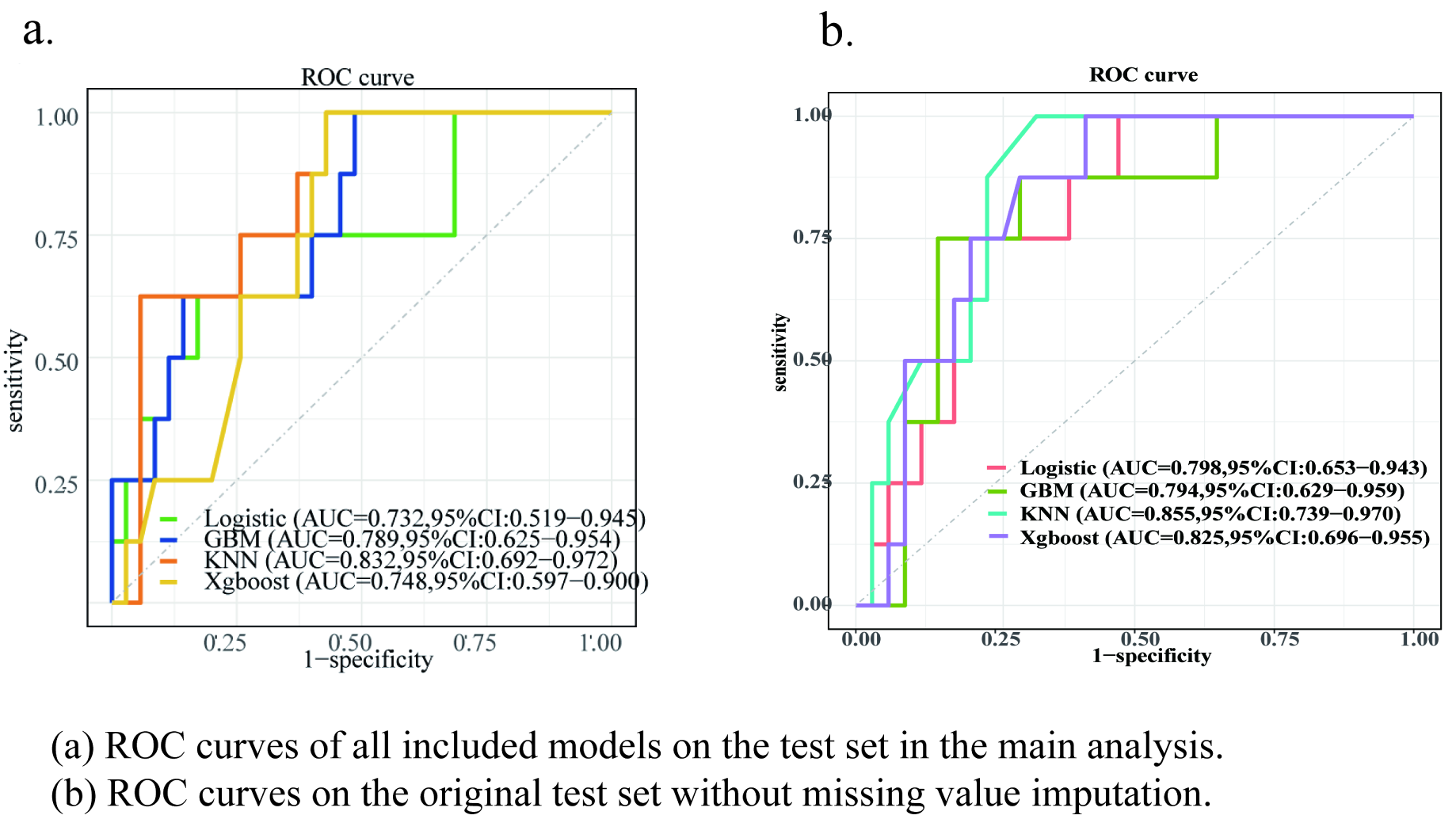

Supplement: Supplementary file 1 [file Image1.tif]
